# Chaperone-Mediated Responses and Mitochondrial–Endoplasmic Reticulum Coupling: Emerging Insight into Alzheimer’s Disease

**DOI:** 10.3390/cells14151179

**Published:** 2025-07-31

**Authors:** Manish Kumar Singh, Minghao Fu, Sunhee Han, Jyotsna S. Ranbhise, Wonchae Choe, Sung Soo Kim, Insug Kang

**Affiliations:** 1 Department of Biochemistry and Molecular Biology, School of Medicine, Kyung Hee University, Seoul 02447, Republic of Korea; manishbiochem@gmail.com (M.K.S.); andrew1179609214@gmail.com (M.F.); sunheehan@khu.ac.kr (S.H.); jogm25@khu.ac.kr (J.S.R.); wchoe@khu.ac.kr (W.C.); 2 Biomedical Science Institute, Kyung Hee University, Seoul 02447, Republic of Korea; 3 Department of Biomedical Science, Graduate School, Kyung Hee University, Seoul 02447, Republic of Korea

**Keywords:** Alzheimer’s disease, amyloid-β, aggregates, calcium, chaperones, endoplasmic reticulum, mitochondria

## Abstract

Alzheimer’s disease (AD) is increasingly recognized as a multifactorial disorder driven by a combination of disruptions in proteostasis and organelle communication. The 2020 Lancet commission reported that approximately 10 million people worldwide were affected by AD in the mid-20th century. AD is the most prevalent cause of dementia. By early 2030, the global cost of dementia is projected to rise by USD 2 trillion per year, with up to 85% of that cost attributed to daily patient care. Several factors have been implicated in the progression of neurodegeneration, including increased oxidative stress, the accumulation of misfolded proteins, the formation of amyloid plaques and aggregates, the unfolded protein response (UPR), and mitochondrial–endoplasmic reticulum (ER) calcium homeostasis. However, the exact triggers that initiate these pathological processes remain unclear, in part because clinical symptoms often emerge gradually and subtly, complicating early diagnosis. Among the early hallmarks of neurodegeneration, elevated levels of reactive oxygen species (ROS) and the buildup of misfolded proteins are believed to play pivotal roles in disrupting proteostasis, leading to cognitive deficits and neuronal cell death. The accumulation of amyloid-β (Aβ) plaques and tau neurofibrillary tangles is a characteristic feature of AD. These features contribute to chronic neuroinflammation, which is marked by the release of pro-inflammatory cytokines and chemokines that exacerbate oxidative stress. Given these interconnected mechanisms, targeting stress-related signaling pathways, such as oxidative stress (ROS) generated in the mitochondria and ER, ER stress, UPR, and cytosolic chaperones, represents a promising strategy for therapeutic intervention. This review focuses on the relationship between stress chaperone responses and organelle function, particularly the interaction between mitochondria and the ER, in the development of new therapies for AD and related neurodegenerative disorders.

## 1. Introduction

Neurodegeneration underlies a spectrum of age-related disorders characterized by progressive synaptic failure and neuronal loss, which ultimately affect memory, executive function, and behavior. This decline culminates in conditions such as Alzheimer’s disease (AD), Parkinson’s disease (PD), amyotrophic lateral sclerosis (ALS), and various forms of dementia, including frontotemporal dementia (FTD), Lewy-body dementia (LBD), and vascular dementia (VaD) [1]. In addition to the more common types of dementia, there are several rare forms, including autosomal dominant Alzheimer’s disease, Huntington’s disease, progressive supranuclear palsy, corticobasal degeneration, Creutzfeldt–Jakob disease, multiple system atrophy, and Wernicke–Korsakoff syndrome [2]. Thirty to fifty percent of all rare diseases affect the nervous system, impacting cognition and overall function. Currently, there are approximately 3.9 million individuals worldwide with early onset dementia [3].

AD is the most prevalent form of dementia and is primarily associated with memory loss and cognitive deficits [1]. A hallmark of AD is the intracellular accumulation of neurofibrillary tangles (NFTs), which consists of hyperphosphorylated tau proteins. Additionally, Aβ is a peptide fragment generated by the proteolytic cleavage of amyloid precursor protein (APP) by the proteases, β-secretase, and γ-secretase. The cleavage of APP by β-secretase (BACE1), followed by α-secretase, produces shorter, soluble peptides, Aβ1–15 and Aβ1–16. However, the cleavage at the C-terminal end of APP by γ-secretase involves both Aβ1-40 and Aβ42 fragments. Both Aβ1-40 and Aβ42 are neurotoxic and are deposited in the hippocampus and cortex in an AD brain, leading to synaptic dysfunction, neuronal death, and cognitive decline [4]. The soluble Aβ oligomers (AβOs) and protofibrils in the brain represent the most pathogenic forms of Aβ, especially since individuals with these forms can develop early dementia even in the absence of core Aβ plaques [5]. In sporadic late-onset AD (LOAD), elevated levels of Aβ42 and the hyperphosphorylation of tau protein are characteristic features of the disorder. The intracellular effects of AβOs may arise from surface mechanisms or could result from direct interactions between organelles and internalized AβOs. Internalized Aβ (monomers and AβOs) is localized to various organelles (ER, Golgi bodies and multivesicular bodies/late endosomes, lysosomes, exocytotic vesicles, and mitochondria ) as well as non-membrane bound cytosolic structures [6,7]. The uptake of Aβ via endocytosis occurs rapidly and spontaneously, and it is retained in lysosomes, where its accumulation can lead to aggregation [8].

**Figure 1:**
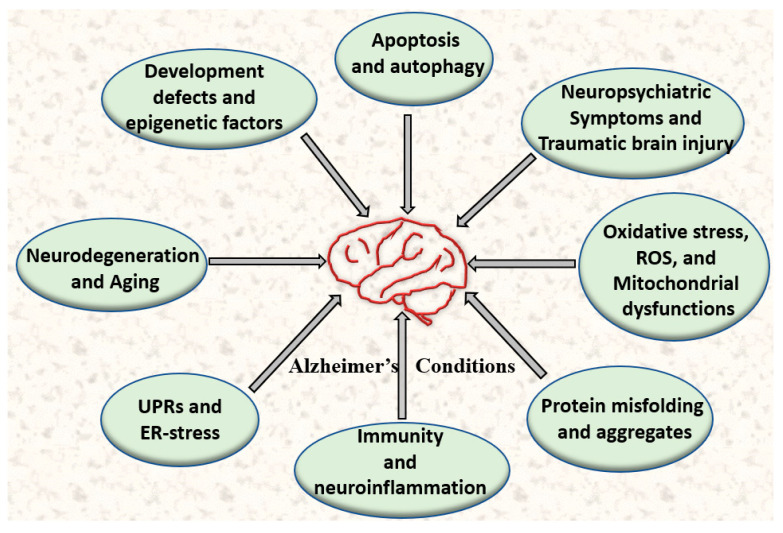
The image illustrates multiple interconnected biological processes that contribute to the progression of Alzheimer’s disease. Key factors involved include oxidative stress (ROS), protein misfolding and unfolding, dysfunction between mitochondria and ER, as well as age-related cellular changes. Together, these processes lead to neuronal damage and cognitive decline in AD.

Multiple converging stressors accelerate the progression of disease. For instance, genetic variation, epigenetic changes, environmental toxins, and chronic psychosocial stress all contribute to increased levels of ROS, lipid peroxidation, and mitochondrial dysfunction (Figure 1). Early AD pathology is closely linked to early life stress and inflammatory signals, which exacerbate Aβ deposition and increase levels of pTau [9]. Activated microglia and astrocytes play a dual role in this context, as they facilitate the clearance of Aβ while also alleviating cognitive dysfunction [10]. However, glial cells, including microglia and astrocytes, also produce and release large amounts of pro-inflammatory cytokines as well as reactive oxygen and nitrogen species (ROS and RNS), which can have detrimental effects on neuronal function and may lead to cell death [11]. Studies indicate that tau phosphorylation, in combination with oligomeric and fibrillar Aβ peptides, elicits a vigorous immune response from microglia in reaction to the early accumulation of synaptotoxic AβOs or emerging tau pathology [12].

Molecular chaperones play a crucial role in protein quality control and are upregulated in response to cellular stress, thereby maintaining cellular proteostasis. Hsps are classified based on their sequence homology and molecular weight, including small heat shock proteins, Hsp60, Hsp70, and Hsp90. Studies have demonstrated that various stresses such as ROS, DNA damage, protein aggregates, and pathological conditions can exacerbate AD pathology [13]. When the capacity of chaperones fails, “chaperonopathies” can arise, contributing to AD and related diseases [14]. Recent research highlights the potential of both cytoplasmic and organelle-specific chaperones, particularly ER- and mitochondrial-resident chaperones as promising yet under-explored therapeutic targets. Extracellular chaperones, co-chaperones, and chaperone-like amyloid-binding proteins (CLABPs) can directly neutralize amyloidogenic peptides. For instance, clusterin, along with Hsp70 and Hsp90, has shown potential in preventing the aggregation of amyloidogenic peptides by interfering with the nucleation reaction and inhibiting fibrillar formation [15,16]. Notably, the molecular chaperone domain BRICHOS contains three proproteins, namely Bri2, Bri3, and ProSP-C, which are ATP-independent molecular chaperones that potentially block Aβ secondary nucleation [17]. In APP/PS1 mice, co-expression or systemic delivery of BRICHOS reduces soluble and insoluble Aβ, abates gliosis, and improves cognitive functions, outperforming the monoclonal antibody aducanumab in vitro [18]. Another example is the mutant nucleobindin-1 (mtNUCB1), which traps protofibrils of Aβ, α-synuclein, and other amyloidogenic proteins, serving as a novel immunogen for conformation-specific antibody discovery [19]. These findings suggest that selective modulation of chaperone—rather than broad anti-aggregation strategies—may be a compelling approach for disease modification. In addition to CLABPs, many other proteins, including proteases, have been identified that can modulate various stages of APP processing, Aβ aggregation, mitochondrial ER stress, and Ca^2+^ homeostasis in AD patients and models (Table 1).

Calcium dyshomeostasis represents another intersecting axis of vulnerability. Precise Ca^2+^ signaling governs neurotransmission and synaptic plasticity; its disruption is an early event in AD, contributing to Aβ accumulation and tau phosphorylation [20]. For intraneuronal Ca^2+^ dyshomeostasis to trigger AD pathology, the perturbation of Ca^2+^ signaling must occur early in the disease process. In the early stages of AD, overactivation of NMDAR serves as a potential source of intracellular calcium, playing a significant role in excitatory synaptic neurotransmission [21]. Memantine, a non-competitive NMDAR inhibitor, is currently approved for the treatment of AD [22]. Disruption of ER Ca^2+^ release leads to mitochondrial stress, overproduction of ROS, caspase-3 activation, and impaired mitophagy in neurons derived from patients. Coordinated targeting of mitochondrial–ER stress pathways, ROS, and Ca^2+^ signaling could synergistically slow the progression of AD. This review discusses current evidence on molecular chaperones, particularly cytosolic Hsps and mitochondrial and ER stress responses, including the unfolded protein response in AD. We highlight emerging chaperone-based interventions and outline how the integrated modulation of proteostasis, ROS, and Ca^2+^ dynamics could yield next-generation therapeutics for AD and other neurodegenerative diseases.

**Table 1:** List of proteins involved in neurodegeneration and regulation of AD disease progression.

**Proteins**	**Function and Location**	**Molecular and Cellular Functions**	**Reference (s)**
Angiotensin-converting enzyme (ACE)	Upregulated in hippocampus	ACE inhibition retards tau hyperphosphorylation and prevents glutamate toxicity via Gad2	[23]
Asparagine endopeptidase (AEP)	Cysteine protease	Generate toxic pTau and Aβ40 and Aβ42 levels in the cytoplasm; inhibition of APE improves cognitive deficits	[24]
Arachidonate 5-lipoxygenase (ALOX5)	Induces expression in hippocampus in AD patients	Induces Tau phosphorylation and activates GSK3β in knockout AD mice model	[25]
Acetylcholinesterase (AChE)	Serine hydrolase and found in neuromuscular junction and cholinergic synapses	Hydrolyzes acetylcholine and is involved in cholinergic neurotransmission; inhibition of AChE improves cognitive deterioration	[26]
Butyrylcholinesterase (BuChE)	Involved in synaptic hydrolysis of ACh	Upregulates in AD and linked with abnormal Aβ production	[27]
Calnexin (CANX)	ER-resident chaperone	Regulates ULK1 dependent autophagy deficiency linked with cognitive function	[28]
Calreticulin (Crt)	ER-resident chaperone, calcium binding protein, binds to glycoproteins	Involved in binding of O-glycosylated form of APP	[29]
Calpains (1 and 2)	Calcium-dependent proteases, upregulate in CNS	Induce p25/Cdk5 activity; upregulate BACE1, PS1, and Aβ formation. Inhibition results in decreased pSTAT3 and mitigates Aβ production	[30]
Cathepsins (B, D, and E)	Lysosomal proteases	Associated with the risk of AD and pTau	[31]
Clusterin (CLU)	Found in plasma and CSF of AD patients	Promotes plaque deposition and AD pathology	[32]
Corticotrophic-releasing factor (CRF) and receptor R1 (CRFR1)	Stress-related protein	Act as mediator of β or γ-secretase activity, expression, and/or trafficking	[33]
Cyclin-dependent kinase 5 (CDK5)	Serine/threonine kinase, overactivation of Cdk5 is conducive to Aβ production and accumulation	Hydrolysis of APP, and promotes the activity and level of β-site APP cleaving enzyme 1 (BACE1) and γ-secretase	[34]
Fyn kinase and N-methyl-D-aspartate (NMDA) subunit NR2B Aβ	Hippocampus and prefrontal cortex	Reelin downregulation impairs Fyn-NMDAR2B-CREB signaling pathway, which leads to cognitive impairment in AD models	[35]
FK506-binding protein 51 (FKBP51)	Co-chaperone of Hsp90; regulates glucocorticoid receptor	Modulates GR activity and Hsp90 levels, blocking FKBP51, and Hsp90 interaction promotes tau pathogenicity	[36,37]
Furin	Golgi, trans Golgi network and endosomes	Furin inhibition reduces cleavage of the BACE propeptide and, thus, modulates Aβ synthesis	[38]
Glycogen synthase kinase-3β (GSK-3β)	Serine/threonine kinase, insulin pathway regulator	Promotes tau hyperphosphorylation, promotes neurofibrillary degeneration, and reduces long-term potentiation (LTP)	[39]
Insulin-degrading enzyme (IDE)	Zinc-dependent metallopeptidase highly expressed in brain	IDE deficiency leads to microgliosis and Aβ deposition	[40]
Myelin basic protein (MBP)	Component of neuromyelin and present in neurons and CSF	Increased in white matter degeneration and general brain atrophy	[41]
Monoamine oxidase (MAO)	Two isoform MAO-A and MAO-B in brain, MAO-B is linked with enhanced generation of free radical and H_2_O_2,_ linked with AD pathogenicity	Catalyzes the oxidation of biogenic amine neurotransmitters such as norepinephrine, dopamine, and 5-hydoxytryptamine (serotonin)	[42]
Matrix Metalloproteinases (MMPs-2, 9 and 10)	Zinc-dependent endopeptidases in reactive astrocytes	Degrade extracellular matrix proteins; cleave amyloid precursor protein (APP) and Aβ into non-toxic fragments	[43,44]
Nuclear receptor Sub-family 4 group A member 1 (NR4A1)	Nuclear receptor with three isoforms in brain	Promotes APP amyloidogenesis via ADAM10 and BACE1 regulation; affects pTau	[45]
Nucleobindin 1	Golgi-resident Ca^2+^ binding protein	Prevents APP-induced cytotoxicity in AD models	[46]
Protein Phosphatase 2A (PP2A)	Serine/threonine phosphatases	Involved in Tau hyperphosphorylation and reduces expression in AD at gene and protein levels	[47]
Subtilisin/Kexin Type 9 (PCSK9)	Negative regulator of brain cholesterol homeostasis and neuroinflammation	Higher level of PCSK9 in the CSF of AD brain; facilitates Aβ clearance via LRP1	[48]
Repressor element 1-silencing transcription (REST)	Neuronally derived exosomes in blood plasma	Modulates neuronal differentiation and contributes to AD pathology and cognitive impairment	[49]
Thrombin	Serine proteases, reported in both in the vessel walls and senile plaques	Involved in cerebrovascular response to hypoxia and oxidative stress (ROS) in brain epithelial cells in AD	[50]
Visinin-like protein-1 (VLP-1)	Present in cerebrospinal fluid (CSF) and upregulated in AD	Modulates intracellular calcium and oxidative stress; elevated in neuronal degeneration	[51]

## 2. Oxidative Stress and Proteostasis in AD

Environmental stressors, such as heat shock, hypoxia, acidosis, and proteotoxic insults, drive a heterogeneous group of normally soluble proteins to co-aggregate into reversible intracellular condensates known as amyloid bodies (A-bodies) [52]. Emerging evidence indicates that A-bodies are not just passive deposits; instead, they are stress-specific assemblies that modulate cellular responses. Each type of stress appears to trigger the selective recruitment of distinct client proteins, resulting in subtype-specific “molecular barcodes.” However, it remains unclear how the regulators of A-bodies influence the transition of disease-linked peptides to pathological amyloid states. One possibility is that local cell death releases A-body components into the extracellular milieu, which could reduce the nucleation lag of nearby aggregation-prone peptides and accelerate fibril formation. Alternatively, prolonged exposure to stress-activated signaling pathways may enhance the recruitment and misfolding of inherently amyloidogenic proteins, thereby promoting the progression of AD [53]. Oxidative stress, which intensifies with age as antioxidant defenses decline and metabolic free radical production increases, interacts with these phase-transition events. Neuronal reactive oxygen species (ROS) include superoxide anion (O_2_^−^), hydrogen peroxide (H_2_O_2_), and the highly reactive hydroxyl radical (HO•), along with reactive nitrogen species such as nitric oxide (NO) and peroxynitrite (ONOO^−^). Moreover, the redox sensitivity of protein cysteine thiols, which responds to redox changes and alterations in total protein levels, may serve as a valuable indicator of oxidative stress and protein modification. Measuring shifts in the cysteine redox pool provides insight into the redox status of proteins and their functional states under disease conditions [54].

Mitochondria are the primary source of ROS, and their cumulative dysfunction contributes to neuronal decline and aging. Manganese-superoxide dismutase (Mn-SOD) in the matrix and Cu/Zn superoxide dismutase (Cu/Zu-SOD) in the cytosol convert O_2_^−^ to oxygen (O_2_) and (H_2_O_2_), which are further detoxified by catalase and glutathione peroxidases. However, transition metal ions (Cu, Fe) can catalyze Fenton chemistry, generating additional free radicals that drive oxidative damage. This can lead to elevated levels of protein carbonyls, DNA oxidation, malondialdehyde, and lipid peroxidation, loss of cytochrome C oxidase, accumulation of advanced glycation end products, and increased ONOO^−^ levels and heme oxygenase -1 [55]. While endogenous antioxidant systems, such as vitamins, thiol antioxidants (lipoic acid, glutathione), and flavonoids, offer protective effects against these insults, chronic ROS overload ultimately destabilizes proteostasis and energy metabolism, priming neurons for amyloidogenesis and synaptic dysfunction.

### Mitochondrial Chaperones, Proteases, and Calcium Regulation in AD

**Figure 2:**
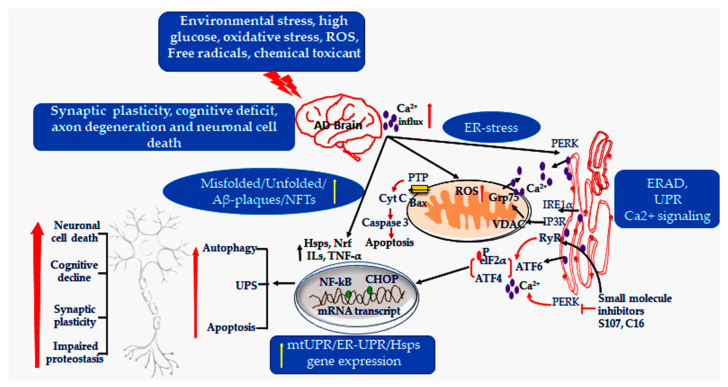
The image illustrates the interactions between ER and mitochondria under AD conditions. Mitochondrial–ER contact sites (MERCS) play a crucial role in regulating various cellular processes, including ER stress, the unfolded protein response (UPR), and protein degradation pathways such as the ubiquitin–proteasome system (UPS), autophagy, and apoptosis. Notably, MERCS are involved in ER–mitochondrial calcium (Ca^2+^) signaling through the mitochondrial calcium uniporter (MCU), and they influence the transcription of genes associated with AD risk as well as the processing of APP. Changes in the coupling between the ER and mitochondria observed in AD disrupt these processes, underscoring their significance in the progression of disease. In the diagram, the symbol (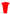) indicates the expression of upregulated proteins, (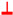) denotes inhibitory activity, and (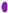) represent the dynamics of Ca^2+^ ions within the pathways associated with ER and mitochondria.

Oxidative stress is a key factor in mitochondrial dysfunction, disrupting the activity of the electron-transport-chain (ETC) and altering the balance between the generation of free radicals and endogenous antioxidant defenses. Elevated mitochondrial ROS can intensify unfolded protein responses (UPRs), potentially leading to either PARK2/Parkin-mediated mitophagy or apoptosis [56]. Moreover, increased ROS levels contribute to the production of Aβ, thereby accelerating the progression of AD pathology. Molecular chaperones play a crucial role in protecting mitochondria from oxidative stress. For instance, reduced levels of mitoHsp60 in the brains of insulin-resistant mice highlight its importance for neuronal energy metabolism [57]. Other mitochondrial chaperones, such as mortalin (mtHsp70) and glucose-regulated protein 75 (Grp75/HspA9), along with tumor necrosis factor receptor-associated protein 1(TRAP1), help mitigate oxidative stress, regulate mitophagy, and manage the fission–fusion dynamics of mitochondria [58]. In AD, the decreased levels of HSP8 and HspA9 may be linked to a reduction in Aβ clearance [59]. Experimental studies suggest that mitochondrial Hsps, including Hsp60, Hsp70, and Hsp90, work both independently and collaboratively to support oxidative phosphorylation and the function of enzymes in the tricarboxylic acid cycle during challenges from intracellular Aβ. Aβ specifically inhibits ETC complex IV, but this effect can be fully reversed by Hsp60 [60]. In vitro studies have shown that mitochondrial Hsp60 serves as a primary response to oxidative stress, and its translocation to the cytoplasm sensitizes neuroblastoma cells to apoptosis [61]. Upregulating these Hsps can reduce cytochrome C release and caspase-9 activation (Figure 2). Conversely, broad inhibition of Hsps with quercetin exacerbates intracellular Aβ accumulation, complex IV failure, and apoptotic death in primary cortical neurons. These findings identify mitochondrial Hsps as promising targets for early intervention in AD.

The mitochondrial matrix peptidase known as human Presequence Protease (hPreP) is reduced due to increased ROS production. This reduction contributes to the accumulation of Aβ in mitochondria, leading to mitochondrial toxicity and neuronal death, which are exacerbated in AD [62]. Therefore, hPreP demonstrates a protective function in the pathology of AD. Mutations in human pitrilysin metallopeptidase 1 (PITRM1), a mitochondrial matrix enzyme, are linked to autosomal recessive spinocerebellar ataxias (ARCA) that occur in early childhood. These mutations disrupt the degradation of long peptide substrates (≥40 amino acids), particularly impacting Aβ metabolism [63]. When PITRM1 is knocked out in cerebral organoids, it activates UPR^mt^, which in turn triggers cytosolic quality control mechanisms such as UPS and autophagy, mimicking similar pathological features observed in AD [64]. Thus, hPreP may offer a potential target for treating AD and inflammatory neurodegeneration. Additionally, the pro-apoptotic Bcl2-associated X (Bax) is found to be upregulated in both AD and ALS, linking it to mitochondrial dysfunction and necrotic cell death [65]. The upregulation of BAX facilitates the release of cytochrome C (Cyt C) from the mitochondria into the cytosol, leading to apoptotic neuronal cell death.

Mitochondrial dysfunction in AD is further intricately linked to disturbed Ca^2+^ signaling. Elevated levels of Ca^2+^ ions in the mitochondrial matrix intensify Aβ toxicity and compromise the efficiency of the ETC [66]. Studies have indicated that increased free Ca^2+^ in the mitochondrial matrix enhances Aβ toxicity [67]. Partial inhibition of the mitochondrial Ca^2+^ uniporter (MCU) using small molecules such as TG-2112x can protect neurons from excitotoxicity caused by glutamate without impairing membrane potential or respiration; it even has the potential to restore cognitive functions in Aβ-treated models [68]. Moreover, research has demonstrated that inhibiting the mitochondrial permeability transition pore (mPTP) can improve cognitive function in transgenic mouse models of AD [69]. Cyclophilin-D (CypD), a key regulator of mPTP, is hyperactivated in AD. Pathological tau exacerbates the sensitivity of CypD to Ca^2+^, while tau knockdown reduces mPTP openings, helping to restore mitochondrial resilience [70,71]. Similarly, reinstating the expression of the Na^+^/Ca^2+^ exchanger NCLX completely reverses cognitive deficits and mitochondrial pathology in AD mice [72]. Compounds that stimulate mitophagy, such as urolithin A and resveratrol, promote the clearance of dysfunctional mitochondria and restore mitostasis (Table 2) [73]. Targeted antioxidants, including coenzyme Q_10_, MitoQ, SkQ1, MitoApo, and astaxanthin, have been shown to reduce ROS levels, enhance ETC performance, and improve cognition in various AD models [74,75,76]. Their localization to the inner mitochondrial membrane enables more efficient scavenging of ROS than untargeted antioxidants. Collectively, these findings highlight the therapeutic potential of targeting mitochondrial dysfunction in AD.

## 3. Molecular Chaperones and Their Role in AD and Neurodegeneration

Neurological disorders are often linked to the misfolding, unfolding, and aggregation of proteins, which lead to the accumulation of these proteins in neurons. Hsps play a crucial role in protein quality control and are classified into the following groups based on their molecular weight: small heat shock proteins (small Hsps), Hsp60, Hsp70, Hsp90, and high-molecular-weight Hsps [77]. Small Hsps and specialized J domain chaperones primarily interact with oligomeric protein species to prevent further aggregation and reduce cytotoxicity. In addition to cytosolic chaperones, there are organelle-specific chaperones, such as mitochondrial chaperones like mortalin (mtHsp70 or Grp75) and TRAP1, as well as ER chaperones like HspA5 (Bip). These chaperones help manage protein quality control and protect cells against oxidative stress, ROS, and various types of toxicity. Moreover, proteins containing the BIRCHOS domain have been shown to prevent the aggregation and oligomerization of amyloidogenic proteins, particularly Aβ42 and Aβ40 [78]. The extracellular chaperone clusterin (CLU) plays a crucial role in sequestering small oligomers into larger, less toxic extracellular aggregates. For instance, CLU-deficient mice have shown that insoluble protein deposits can accumulate in the kidney, leading to progressive glomerulopathy [79]. These findings indicate that CLU is essential for clearing misfolded proteins from the extracellular environment, although the underlying mechanisms involved remain unclear. In summary, among the various molecular chaperones, Hsps play a vital role in preventing the misfolding, unfolding, and aggregation of proteins in neurons, including amyloidogenic proteins. They also facilitate the clearance of aggregated and misfolded proteins within the neurons [80].

### 3.1. Small Hsps Role in AD and Neurodegeneration

Small Hsps/HspBs are a family of cytosolic chaperones with molecular weights ranging from 14 to 43 kDa. In mammals, ten distinct small Hsps have been identified [81]. Among these, HspB2 is highly expressed in cardiac and skeletal muscle and plays a role in metabolic regulation and mitochondrial energetics [82]. Hsp27 (HspB1) is the most extensively studied neuronal small Hsp in neurons. Its expression increases in both neurons and glial cells during various neurodegenerative disorders, reflecting the accumulation of misfolded proteins associated with these diseases. Beyond its well-documented pro-apoptotic and anti-apoptotic activity, Hsp27 has multiple neuroprotective functions. In AD and multiple sclerosis, elevated levels of Hsp27 are observed alongside increased levels of the related small Hsp, αB-crystallin [83]. Phosphorylated Hsp27 inhibits tau aggregation, accelerates the clearance of tau fibrils, and helps restore long-term potentiation (LTP). It interacts with early tau species through weak, transient connections, preventing the growth of fibrils and facilitating the conversion of toxic Aβ oligomers into larger, innocuous aggregates [84]. Although these effects are well established, the specific molecular mechanisms by which Hsp27 recognizes Aβ remain to be fully explained. The overexpression of Hsp27 provides significant resistance to high temperature and oxidative stress, functioning in a glutathione-dependent manner [85]. These cytoprotective properties are attributed to its chaperone activity, the direct inhibition of caspase activation, the modulation of the cellular redox state, and the regulation of cytoskeletal dynamics. Consequently, Hsp27 is an attractive target for therapeutic interventions in neurodegenerative disease.

Polymorphisms in the genes encoding Hsp22 and Hsp27 have been linked to motor neuron neuropathies [86], while pathogenic mutations of Hsp27 are associated with Charcot–Marie–Tooth disease and distal hereditary motor neuropathy [87]. Recombinant Hsp27 has been shown to alter the quaternary structure of Aβ in vitro, thus reducing its oligomer toxicity. Hsp27 switches between phosphorylated and non-phosphorylated states, facilitating the clearance of abnormal tau and restoring LTP in AD mouse models [88]. Neurons that overexpress HspB1 demonstrate selective protection from Aβ toxicity, resulting in reduced degeneration, preservation of mitochondrial morphology, and enhanced neurite outgrowth in cortical cultures [89]. Other small Hsps also provide significant neuroprotection. For instance, overexpression of Hsp22 (HspB8) in tau transgenic mice improves cognition and synaptic plasticity by modulating key pathways and upstream regulators of synaptogenesis, including EIF4E and NFKBIA, both of which are emerging biomarkers for AD [90]. Additionally, upregulating HSPB8 along with its co-chaperone BAG3 further enhances the ability of astrocytes to remove neuron-derived protein aggregates and cellular debris, thereby supporting local tissue homeostasis [91]. In summary, small Hsps play a crucial role in neuronal differentiation, survival, and repair. Small molecules that can safely induce Hsp27 or broadly enhance small Hsp activity under physiological conditions show significant promise as therapeutics for neurodegenerative diseases and for promoting nerve regeneration.

### 3.2. Hsp60 Role in AD and Neurodegeneration

Hsp60 is primarily located in mitochondria, and its expression significantly increases under oxidative stress. Once induced, some Hsp60 is exported from neuronal mitochondria within exosomes and released into the extracellular environment. Extracellular Hsp60 activates innate immune signaling in both neurons and glial cells by interacting with toll-like receptors (TLRs) 2 and 4. However, some studies suggest that TLR-3 may be the main sensor for Hsp60-driven neuroinflammation [92]. The release of Hsp60 relies on lipid raft-dependent endocytosis, followed by its packaging into exosomes. Increased circulating or tissue levels of extracellular Hsp60 have been observed in various inflammatory diseases, including cancer, diabetes, atherosclerosis, rheumatoid arthritis, insulitis, and several neuroinflammatory disorders, and it can be detected in blood, saliva, and urine [93,94,95]. Interest in exosome-borne proteins has grown in the context of neurodegeneration. The secretion of exosomes within the central nervous system is regulated by glutamatergic activity and Ca^2+^ influx [96]. Exosomes derived from astrocytes can transport prion proteins, enhancing neuronal resistance to oxidative stress [97]. However, they can also carry misfolded pathogenic proteins and microRNAs that exacerbate neuroinflammation and neurodegeneration [98].

Exosomal Hsp60 specifically binds to astrocytic TLRs, triggering pro-inflammatory signaling cascades that lead to the production of proinflammatory mediators such as TNF-α, IL-1β, IL-6, and IL-8 [99]. Additionally, upregulation of Hsp60 in neurons may serve as a potential mediator of neuroinflammation [100]. Profiling the microRNA cargo of neuron-derived exosomes in AD could reveal novel strategies to slow disease progression. The t-complex polypeptide (TCP-1), which shows sequence homology to Hsp60, and its specific substrate β-1 tubulin are significantly reduced in the temporal, frontal, and parietal cortex as well as in the thalamus of patients with AD. This reduction may contribute to the misfolding of cytoskeleton proteins and the neuropathological changes observed in AD, such as plaques and tangles [101]. Notably, Hsp60 interferes with Aβ1-42 fibrillation by disrupting key steps in the pathological aggregation of proteins. Understanding how chaperones counteract these aggregation processes could serve as a foundation for developing novel therapeutic approaches for neurodegenerative disorders [102]. Several studies have reported a strong association between mitochondrial Hsp60 and APP/Aβ in mitochondria isolated from both AD mouse models and human patients [103]. Additionally, knocking down Hsp60 using a viral-mediated shRNA approach has confirmed its essential role in mitochondrial translocation of APP/Aβ [104]. However, current knowledge about extracellular Hsp60 in the context of AD remains limited, highlighting the urgent need for further research.

Moreover, the elevated expression of Hsp60, whether alone or in combination with other heat shock proteins such as Hsp70 or Hsp90, can influence key stages of mitochondrial apoptosis, protecting cells from Aβ-induced toxicity [105]. Interestingly, the isolated apical domain of Hsp60 (Hsp60-AD-Cys) has been shown to suppress α-synuclein fibrillization, acting as a “mini-chaperone” within cells and demonstrating therapeutic potential for Parkinson’s disease and related proteinopathies [106]. The multifunctional nature of Hsp60, driven by its diverse subcellular localizations, has led to its classification as a “moonlighting protein,” a subclass of proteins that perform multiple physiologically relevant functions within a single polypeptide chain [107]. A deeper understanding of these moonlighting roles could pave the way for the development of innovative therapeutic strategies targeting AD and other neurodegenerative conditions.

### 3.3. Hsp70 Role in AD and Neurodegeneration

Hsp70 is constitutively expressed in brain tissues and neuronal cells, playing a vital role in maintaining protein homeostasis against a variety of pathological challenges, including traumatic injury, metabolic and oxidative stress, excitotoxicity, and ischemia [108,109]. An age-related decline in proteostasis—evidenced by reduced proteasomal activity and diminished chaperone capacity—accelerates protein misfolding and aggregation, contributing to neurodegeneration [108]. Hsp70 is essential for sequestering Aβ and other unfolded and misfolded proteins, thereby mitigating their harmful effects. Experimental upregulation of Hsp70 has been shown to reduce α-synuclein aggregation and toxicity by ~50%, aiding both refolding and degradation pathways [110]. In AD, Hsp70 induction has been observed in vulnerable neurons and neighboring astrocytes. The processing of the APP within the ER depends on the ER-resident Hsp70 paralogue Grp78, which supports proper refolding and neuronal viability [111]. Hsp70 also inhibits α-synuclein fibrillization in an ATP-dependent “holdase” mode, stabilizing on-pathway intermediates and preventing the formation of pathogenic aggregates. While α-synuclein interacts with the canonical substrate-binding domain (SBD) of Hsp70, emerging evidence indicates additional non-canonical interaction sites, the structural details of which are yet to be fully understood [112].

Protein aggregation further disrupts synaptic architecture and memory functions in AD, a process exacerbated by a shift from the dynamic Hsp70/Hsp90 “chaperome” network to more rigid, maladaptive “epichaperomes.” Small-molecule inhibitors that disrupt the Hsp90–Hsp70 epichaperome, such as PU-H71 and PU-AD, have been shown to restore synaptic connectivity performance in the PS19 tauopathy model [113]. These compounds thus represent promising strategies for modulating protein homeostasis networks in neurodegenerative disease. Similarly, in PD, the loss of dopaminergic neurons is mitigated by interventions such as dietary restriction or 2-deoxy-D-glucose, which upregulate Hsp70 and Grp78, providing significant neuroprotection in multiple PD models [114]. Beyond its role in refolding, Hsp70 collaborates with co-chaperones like CHIP and parkin to target terminally misfolded proteins for ubiquitin-mediated degradation [115]. Stress-responsive transcription factors (e.g., STAT3 and NF-κB) can stabilize an Hsp90-centric chaperome around tyrosine hydroxylase; pharmacological disruption of this complex using PU-H71 promotes axonal regeneration in vivo [116].

Hsp70 counters Alzheimer-linked proteotoxicity on multiple fronts; it blocks Aβ aggregation, redirects APP processing, and clears Aβ oligomers via the proteasome [117]. Chaperone interactions also extend to prionopathies, where the Hsp70 co-chaperone Hsp40 suppresses pathogenic prion aggregation [118]. Native prion proteins, which typically adopt an α-helical conformation, are essential for cellular functions such as signaling, copper metabolism, redox regulation, and neuronal protection [119]. However, their misfolding into β-sheet-rich structures makes them protease-resistant, leading to excessive aggregation and neurotoxicity [120]. Grp78 facilitates the degradation of these misfolded prion proteins through the proteasomal pathway, highlighting its critical role in maintaining proteostasis [121]. Several small molecules that bind allosteric pockets in either the nucleotide-binding domain (NBD) or SBD of Hsp70 are being repurposed from oncology to neurodegeneration [122]. Rhodacyanine derivatives, such as MKT-077 and YM-01, have been shown to lower tau levels in vitro and ex vivo, while the blood–brain-permeant analogue YM-08 more effectively reduces both total and phosphorylated tau [123,124]. Phenothiazines like methylene blue and Azure C also lower tau species by inhibiting Hsp70 ATPase activity, though with limited selectivity [125]. Additional Hsp70 modulators—including geranylgeranyl acetone, celastrol, and YC-1—and another neurotrophic compound such as J147 are currently under active evaluation. Notably, this neurotrophic compound enhances cognitive performance and upregulates nerve-growth factors in AD models [126]. Collectively, these findings underscore Hsp70 as a versatile therapeutic target for AD and other tauopathies.

### 3.4. Hsp90 Role in AD and Neurodegeneration

Hsp90 is an ATP-dependent, highly conserved chaperone that plays a crucial role in maintaining proteostasis in eukaryotic cells, directing processes related to protein folding, quality control, and signaling pathways [127,128]. It constitutes ~1–2% of total cellular protein, and comprises the following four main isoforms: the inducible cytosolic Hsp90α (Hsp90AA1), the constitutive cytosolic Hsp90β (Hsp90AB1), the ER-resident Grp94, and the mitochondrial TRAP1 [129,130]. Additionally, an alternative splice variant, Hsp90N, is associated with cellular transformation. TRAP1 shares similarities with the bacterial homolog HtpG [131]. The Hsp90 monomer consists mainly of the following three domains: an N-terminal ATP-binding domain, a flexible charged linker, a middle domain that interacts with client proteins and co-chaperones, and a C-terminal dimerization domain [132,133,134]. The middle domain (M) contains a highly charged hinge region (amino acids 206–287), which is essential for binding client proteins and interacting with the co-chaperone. This region enhances substrate affinity and is critical for the chaperone’s activity [135]. Notably, this hinge region is absent in bacterial HtpG, but its evolutionary divergence remains unexplored [136]. The middle domain is essential for facilitating the interaction with co-chaperones and client proteins [135]. The C-terminal domain is responsible for the Hsp90 dimerization. Although the N- and C-terminal regions can individually prevent the aggregation of fully denatured proteins, the full-length Hsp90 is necessary for refolding partially unfolded substrates [137]. While both yeast and human Hsp90 share structural similarities, yeast Hsp90 has a shorter N-terminal domain and a shorter linker compared to hHSP90, which has a more flexible structure, allowing it to spend more time in an open state. This flexibility enables the chaperone to accommodate a broader range of client proteins and co-chaperones, providing additional regulatory mechanisms [138]. Hsp90 has primary functions that assist in the correct folding of newly synthesized proteins and that help stabilize them. It interacts with numerous client proteins, with co-chaperone requirements varying depending on the specific client. Most of the client proteins are involved in signal transduction and are recruited into complexes with Hsp90 via multiprotein Hsp90/Hsp70-based chaperone machinery [139]. Techniques like single-molecule fluorescence and force spectroscopy refine our understanding of these interactions [140].

Age-related upregulation of several Hsp90 co-chaperones contributes to proteotoxic stress. Extracellular Hsp90 modulates immune responses by activating phagocytes and the Toll-like receptor 4 (TLR4) pathway, promoting the clearance of Aβ, and the phosphorylation and dephosphorylation of tau through stabilization of tau kinases [13]. Despite this, many co-chaperones like pp5, Cdc37, and CacyBP/SIP phosphatase can dephosphorylate tau [141,142]. CHIP is also involved in the degradation of improperly aggregated tau through ubiquitination. A reduced level of CHIP leads to the pathological accumulation of tau, and impairment of STI1/Hop is shown to contribute to tauopathy. These findings suggest that a decline in co-chaperone function due to aging may be a factor in neurodegenerative conditions such as AD [143]. However, under pathological conditions, Hsp90 activity may facilitate tau aggregation, making Hsp90 inhibition a potential strategy to reduce tau levels and lower Aβ toxicity [144]. Hsp90 inhibitors are broadly categorized into N-terminal and C-terminal inhibitors. N-terminal inhibitors, such as geldanamycin and its less toxic analog 17-AAG, promote tau clearance by disrupting ATP binding. Other N-terminal inhibitors based on purine scaffolds (e.g., EC102, PU24FCI) have also shown efficacy in reducing tau levels. For instance, in the Tg2576 mouse model and cultured neurons, 17-AAG has been shown to mitigate damage caused by soluble Aβ and enhance the expression of synaptic proteins via activation of HSF1 [145]. Similarly, radicicol has shown comparable neuroprotective effects and is considered a potential therapeutic agent for neurodegenerative diseases [146]. C-terminal inhibitors—such as celastrol, novobiocin, and its derivatives KU-32 and A4—exert neuroprotective effects by modulating interactions with client proteins without inducing the heat shock response [147].

Proteins such as ATPase homolog 1 (AHA1), the peptidyl-prolyl cis-trans isomerase FKBP51, and Hsp-organizing protein (HOP) are overexpressed with aging and contribute to the disease progression. Overexpression of AHA1 accelerates tau fibrilization, while FKBP52 interferes with the binding of AMPA receptors, which are essential for synaptic plasticity [148,149]. Inhibition of AHA1, using KU-177, reduces tau toxicity in AD models [150]. Elevated FKBP51 skews the Hsp90 complex toward tau pathology, an effect that can be reversed by an allosteric Hsp90 activator, LA1011 [36,37]. Additionally, overexpression of HOP amplifies α-synuclein toxicity in Parkinson’s disease and amyloid toxicity in AD [151,152]. Inhibition of cytosolic Hsp90 triggers compensatory induction of Hsp70, which may be neuroprotective. For instance, 17-AAG improves synaptic function in AD mice [145], while SNX-0723 has been shown to reduce α-synuclein toxicity in PD rats [153,154]. Disrupting the Hsp90–Cdc37 scaffold using compounds like celastrol, withaferin A, platycodin D, or kongensin A destabilizes neurotoxic kinase complexes [155,156,157,158,159]. Several pan-Hsp90 inhibitors have advanced to phase II clinical trials; however, these treatments often lead to dose-limiting hepatotoxicity and ocular toxicity due to their non-selective targeting of isoforms [160]. Therefore, the development of isoform-selective or co-chaperone-directed modulators could provide a promising approach to harnessing Hsp90 biology while reducing the adverse effects associated with neurodegenerative disease.

### 3.5. Ubiquitin and Its Role in Neurodegeneration

The ubiquitin–proteasome system (UPS) is the primary mechanism for regulated protein turnover in eukaryotic cells [161]. Ubiquitination, an essential post-translational modification, targets thousands of substrates and regulates various processes, such as transcription, cell-cycle progression, and stress responses. Ubiquitin (Ub), a protein consisting of 76 residues (~ 8 kDa), can be appended as either mono- or poly-Ub chains. The latter are recognized and degraded by the 26S proteasome [162,163,164]. Ubiquitination occurs through a three-enzyme cascade involving E1 (activating), E2 (conjugating), and E3 (ligating) ubiquitinases [165]. Deubiquitinases (DUBs) reverse this signal, thereby fine-tuning proteostasis and cellular signaling.

There are nearly 100 human DUBs, classified into the following seven families: UCH, USP, OTU, Josephin, JAMM/MPN+, MINDY, and ZUFSP [166,167,168]. The activity of DUBs is highly specific and is regulated at multiple levels to differentiate between various ubiquitin-like molecules, isopeptides, linear peptides, and different types of ubiquitin linkages and chain structures. For instance, Usp29 binds to and cleaves poly-Ub chains from p53 under oxidative stress [169], whereas inhibiting USP14 accelerates the degradation of multiple proteasome substrates [170,171]. Unwanted proteins are targeted by chaperone-bound ubiquitin E3 ligases, such as STIP1 homology and u-Box containing protein 1 (CHIP), BAG cochaperone 1 (BAG1), and scythe [172]. The ER-associated degradation (ERAD) process relies on HRD1 (HMG-CoA reductase degradation 1) or Gp78 (AMFR), along with the adaptor protein SEL1L, to assist in substrate recognition. E2 enzymes UBC6/7, linked with E3 ligases and the AAA-ATPase p97/VCP, extract ubiquitinated substrates from the ER membrane. Deubiquitinating enzymes, including USP19, regulate ERAD by removing the ubiquitin tag from proteins. ERAD also utilized Derlin-1/2/3 and the Sec61α translocon to retrotranslocate misfolded clients, with USP19 trimming Ub chains to modulate turnover.

Dysfunction of UPS is associated with neurodegeneration. In AD, activation of UPR promotes tau phosphorylation and aggregation of Aβ peptides [173]. The failure of ER proteostasis leads to the accumulation of aberrant proteins, which are subsequently targeted for degradation via either the autophagy–lysosome system (ALS) or the UPS. As AD progresses, the accumulation of pathological proteins, including Aβ and hyperphosphorylated tau (pTau), becomes more pronounced in the brain. The UPS plays a crucial role in degrading misfolded proteins under ER stress [174]. Impairment of the UPS and calcium homeostasis induces ER stress, exacerbating the progression of AD [175]. Notably, acute ER stress upregulates ERAD-associated chaperones, which enhance the UPS-mediated clearance of misfolded or unfolded APP, thereby preventing Aβ production [176]. Despite these findings, the mechanistic relationship between APP processing and the UPS pathway under acute ER stress remains poorly defined and warrants further investigation.

## 4. Endoplasmic Reticulum Stress in AD

The ER plays a vital role in protein folding, lipid biosynthesis, and Ca^2+^ storage in eukaryotic cells [177]. As we age, neurons lose their ability to maintain protein homeostasis, leading to the accumulation of improperly ubiquitinated, oxidized, or misfolded proteins within the ER lumen [178]. When the amount of unfolded proteins surpasses the ER’s capacity to fold them, it triggers increased ER stress. To counteract this stress, the UPR is activated as a protective measure. This response enhances the production of molecular chaperones, reduces overall protein synthesis, and promotes ER-associated degradation (ERAD). ERAD is a process that retrotranslocates misfolded proteins back to the cytosol for degradation via UPS or lysosomal pathways [179]. Proteins that escape ERAD are eliminated through selective autophagy of the ER, known as ER-phagy [180]. However, when ER stress persists for an extended period or remains unresolved, the UPR can switch from a protective role to a pro-apoptotic one, contributing to synaptic dysfunction and neurodegeneration [181,182]. In AD, the accumulation of unfolded proteins, coupled with disrupted Ca2+ homeostasis, worsens oxidative stress and metabolic disturbances. This intensifies ER stress, leading to neuronal cell death. Chronic ER stress can also activate inflammatory pathways and trigger either apoptotic or autophagy responses in AD [183]. A thorough understanding of ER stress, the UPR, and related molecular mechanisms, including ER-resident proteases, ER-Ca^2+^ signaling, and MERCS, is essential. This knowledge will assist in identifying new therapeutic targets and developing more effective interventions for AD and other neurodegenerative disorders.

### 4.1. Endoplasmic Reticulum Associated Degradation (ERAD) in AD

ERAD is a highly conserved protein quality control pathway that targets misfolded proteins in the ER for proteasomal degradation in cytosol [184]. The formation of Aβ plaques and aggregates is associated with the misfolding or unfolding of proteins, leading to their accumulation in the brain. When misfolded proteins accumulate in the ER, they trigger ER stress, which activates both the UPR and ERAD pathways [185]. Within the ER lumen, molecular chaperones and folding enzymes such as BiP/Grp78, protein-disulfide isomerase (PDI), calnexin, and calreticulin facilitate the proper folding and glycosylation of nascent secretory and membrane proteins (Table 1) [186]. Correctly folded proteins are transported to the Golgi apparatus via vesicles, while misfolded proteins are either refolded or directed for degradation through the ERAD pathway [187].

Misfolded proteins are recognized within the ER lumen or through transmembrane domains (TMDs) and are retrotranslocated to the cytoplasm for degradation. This retrotranslocation is mediated by the ATPase complex VCP/p97, which directs substrate proteins to the cytoplasm for proteasomal degradation [188]. Several putative ERAD complexes have been identified based on their association with E3 ligases, including HRD1, autocrine motility factor receptor (AMFR/GP78/RNF45), membrane-associated RING-CH-type finger 6 (MARCH 6/TEB4), RING finger protein 139 (RNF139/TRC8), RNF5 (also known as RMA1), RNF170, RNF185, and transmembrane protein 129 (TMEM129) [189]. Among these, the SEL1L-HRD1 ERAD complex is the most conserved and well-characterized [190]. Recent studies have shown that SEL1L forms a complex with the ER-resident E3 ubiquitin ligase HRD1, which plays a crucial role in the degradation of abnormal proteins in the ER. Inactivation of SEL1L in mouse neurons results in significant weight loss, severe motor dysfunction, brain atrophy, degeneration of Purkinje and hippocampal cells, and ultimately death. These findings highlight the importance of the ERAD in maintaining ER homeostasis and ensuring neuronal function and viability under physiological conditions [191].

Further mechanistic studies are needed to elucidate how ERAD modulates Aβ production and contributes to AD pathogenesis. Understanding the interplay between ERAD and UPR pathways may provide critical insights for therapeutic interventions targeting proteostasis in AD.

### 4.2. ER Stress Responses and Calcium Signaling in AD

Aβ plaques and hyperphosphorylated tau (pTau) are central to the pathology of AD. The accumulation of tau disrupts protein quality control in the ER, blocking ER-associated degradation (ERAD) and provoking a sustained UPR [192]. PDIs within the ER are crucial enzymes that facilitate proper protein folding by catalyzing the formation of disulfide bonds between cysteine residues. In the ER lumen, PDIs facilitate both disulfide bond formation and isomerization, ensuring correct protein folding. Emerging data suggest that neurons secrete PDIs—most notably PDIA1 and PDIA3—in response to tau pathology. PDIA1 levels are selectively elevated in the cerebrospinal fluid (CSF) of AD patients but not in those with tau-negative dementias. This indicates its potential use as a fluid biomarker for therapies targeting tau or modulating the UPR [193]. Structurally, PDI contains four thioredoxin-like domains (a-b-b′-a′), and the a-type domains have been shown to block Aβ fibrillization in vitro, highlighting the potential of PDI-derived peptides as anti-aggregate therapeutics [194]. Additionally, small-molecule PDI inhibitors, such as E64FC26, exhibit anti-inflammatory activity in rheumatoid arthritis [195]. The chaperone-like functions of PDI for select substrates also warrant further investigation in vivo.

Prolonged ER stress also triggers the UPR pathway, which mitigates ER stress through the following three sensors: inositol-requiring enzyme 1(IRE1), activating transcription factor 6α (ATF6), and PKR-like ER kinase (PERK). Genome-wide association studies have implicated UPR sensors as risk loci for AD. Under normal conditions, these sensors are bound to BiP/GRP78, an ER chaperone [196]. Upon ER stress, BiP dissociates, leading to UPR activation. Activation of IRE1α initiates its endoribonuclease activity, splicing the mRNA that encodes X-box binding protein 1 (XBP1). The spliced XBP1 promotes the transcription of genes that help restore ER proteostasis [197]. Deletion of IRE1 in the CNS reduces Aβ burden and plaque formation in mice, while pharmacologic inhibition of IRE1 worsens ER stress, leading to increased accumulation of amyloid precursor protein (APP) in the ER [198]. Conversely, increased expression of XBP1 in the hippocampus is associated with reduced Aβ deposition and improved synaptic plasticity and memory function [199].

Similarly, PERK activation leads to phosphorylation of eIF2α after dissociation from BiP, which attenuates global protein translation during ER stress. Studies have shown that eIF2α phosphorylation increases BACE1 levels, thereby increasing Aβ synthesis in neurons. Additionally, eIF2α is a well-known negative regulator of synaptic plasticity and learning and memory processes. Inhibition of the PERK/eIF2α pathway in the APP/PS1 AD mouse model enhances synaptic function and cognitive performance [196,200]. ATF6 exists in two isoforms, ATF6α and ATF6β. Upon ER stress conditions, BiP dissociates from ATF6α, allowing it to translocate to the Golgi apparatus, where it is cleaved by site-1 and site-2 proteases, subsequently entering into the nucleus. The N-terminal fragment, ATF6f, functions as a transcription factor that activates UPR target genes overlapping with those regulated by XBP-1 and ATF-4. During acute ER stress, ATF4 collaborates with the C/EBP-homologous protein (CHOP/GADD153) to decrease ER chaperone transcription, promoting cellular adaptation and neuronal survival [201]. Conversely, ATF4 can induce a pro-apoptotic program in the chronic phase, evoking aberrant signals that lead to autophagy and inflammation [202]. Another downstream effector of ATF4 is GADD34, also known as PPP1R15A, which counteracts PERK activity by dephosphorylating elF2α through protein phosphatase 1 (PP1), thereby reinitiating protein folding and translation [203]. This transcriptional program helps restore ER homeostasis in neurons [204]. Notably, ATF6 expression is downregulated in AD models, and its knockdown reduces APP expression while also impairing spatial memory [205,206].

Additionally, calcium signaling defects are intricately linked to the pathogenesis of AD. Calcium overload in neurons can lead to dendrite disintegration, and changes in neuronal morphology depend on the presence and proximity of senile plaques. Deregulation of ER Ca^2+^ is associated with presenilin (PS)-mediated Ca^2+^ leak and/or enhanced Ca^2+^ release through inositol 1,4,5-trisphosphate receptors (IP3Rs) and Ryanodine receptors (RyRs). Familial AD-linked mutations in PS1/PS2 reduce capacitive Ca^2+^ entry (CCE), underscoring the role of presenilin in controlling intracellular calcium homeostasis [207,208]. GWAS meta-analysis has identified the ER-localized RYR3 as an AD risk gene [209]. Extracellular Aβ also elevates intracellular Ca^2+^ levels by inducing oxidative membrane damage. Aβ-Fe^2+^/Cu^+^ complexes generate reactive species that peroxidize lipids, leading to the production of toxic lipid aldehyde, inactivating ion pumps, disrupting the transport of glutamate and glucose, and promoting Ca^2+^ influx [210]. Additionally, the imbalance of ER Ca^2+^ is exacerbated by reduced activity of sarco-endoplasmic reticulum Ca^2+^-ATPase (SERCA) and stress-induced expression of the truncated SERCA1 variant S1T, which is up-regulated via PERK–eIF2α–ATF4–CHOP signaling (Figure 2) [211]. Furthermore, chronic energy deprivation in vivo increases levels of eIF2α and BACE1, exacerbating amyloid pathology in the Tg2576 mouse model [212]. The amplification of S1T contributes to ER stress and mitochondrial apoptosis, increases BACE1 expression, and accelerates the amyloidogenic processing of APP [213]. Store-operated Ca^2+^ entry (SOCE) mediated by stromal interaction molecule (STIM), ORAI channels, and transient receptor potential canonical (TRPC) components is also disrupted in AD [214]. Finally, destabilization of the RyR2 complex, caused by PKA phosphorylation and loss of calstabin-2, leads to elevated cytosolic Ca^2+^ levels. A specific small molecule, S107, has been shown to restore RyR2 stability and normalize Ca^2+^ signaling in AD models [215]. Collectively, these findings highlight the interconnected roles of ER proteostasis factors (e.g., PDIs) and Ca^2+^-handling pathways (e.g., presenilin, SERCA/S1T, SOCE, RyRs) as potential therapeutic targets for AD.

### 4.3. ER–Mitochondrial Interaction in AD

In AD, the interaction between the ER and mitochondria is facilitated by mitochondria-associated ER membranes (MAMs), which play a crucial role in maintaining cellular homeostasis. In yeast, the contact between the ER and mitochondria is orchestrated by the ER–mitochondria encounter structure (ERMES). This structure consists of several proteins, including Mdm10, Mdm12, Mdm34, and Mdm1, as well as the ER-resident membrane protein Mmm1 and the following three peripheral membrane proteins: Mdm34, Mdm1, and Gem1 [216]. Although mammalian homologs of ERMES have not been identified, analogous tethering mechanisms are mediated by different molecular machinery in mammalian cells. A significant player in this process is the phosphofurin acidic cluster sorting protein 2 (PACS2), which is essential for maintaining ER–mitochondria contact. When PACS2 is knocked down, the number of mitochondria ER contact sites (MERCS) decreases, and the transfer of calcium (Ca^2+^) between these organelles is impaired [217]. Similarly, the conditional ablation of mitofusin 2 (Mfn2)—a protein that anchors to both ER and mitochondrial membranes—disrupts MERCS in both in vivo and ex vivo models, as demonstrated by Ca^2+^ imaging experiments [218,219]. Recent studies have shown that, in neurons derived from familial AD (FAD) mutant APP-expressing human neural progenitor cells, the assembly of MERCS is enhanced by the sigma-1 receptor (S1R). This enhancement increases the trafficking of palmitoylated APP to the plasma membrane, where it is cleaved by β-secretase, leading to increased production of Aβ at neuronal processes [220]. However, the MERCS proteome remains incompletely explored, highlighting a key gap in our understanding of MERCS physiology. These findings suggest that MERCS play a significant role in regulating Aβ turnover at synaptic terminals.

Additionally, lipid dysregulation at MERCS appears to contribute to AD pathology. During the early stages of AD progression, an accumulation of lipid droplets and raft-like membrane platforms has been observed. ApoE4, the strongest genetic risk factor for late-onset AD, has been shown to enhance MERCS formation and facilitate the transfer of Ca^2+^ from the ER to mitochondria via the IP_3_R–GRP75–VDAC axis, an effect that disappears when GRP75 is knocked down (Figure 2) [221]. This hyperactive Ca^2+^ flux may lead to mitochondrial Ca^2+^ overload and dysfunction [222]. Moreover, FAD-linked PS2 mutations impair mitochondrial Ca^2+^ uptake, potentially promoting neuronal excitotoxicity [223]. Collectively, these findings underscore MERCS as a crucial point for calcium signaling, lipid metabolism, and Aβ processing in AD. Therapeutically targeting MERCS with small molecules or natural compounds has emerged as a promising strategy to restore communication between organelles and mitigate neurodegenerative processes (Table 2) [224].

### 4.4. The Endosomal–Lysosomal Pathway and AD

Aβ peptides are produced through the sequential proteolytic cleavage of APP in various intracellular compartments, including the endoplasmic reticulum (ER) and the medial and trans-Golgi complex. Aβ40 is predominantly generated in the trans-Golgi apparatus, while Aβ42 is primarily formed in the ER and then transported to the Golgi via secretory vesicles [225]. These Aβ peptides can target mitochondria and lysosomes both intra- and extracellularly. The accumulation of Aβ within mitochondria disrupts the ETC and induces the production of ROS, contributing to cellular oxidative stress. Another key modulator of the autophagy–lysosome pathway is transcription factor EB (TFEB) [226,227]. In AD, reduced levels of nuclear TFEB have been observed in the brains of AD patients and aged experimental models [228]. Interestingly, increased expression of TFEB downstream genes has also been noted in the brains of AD patients. Although TFEB mRNA does not change in cortical neurons, its expression increases in hippocampal tissues. Therefore, modulating TFEB expression through pharmacological or genetic approaches may offer an opportunity to halt or even reverse the progression of AD, potentially mitigating its pathology [229].

Genetic mutations, particularly in presenilin-1 (PSEN1), are known to cause early onset AD by impairing lysosomal proteolysis and autophagy [230]. PSEN1 mutations disrupt V-ATPase-mediated lysosomal acidification, which leads to calcium dyshomeostasis in the lysosome [231]. Calcium released from the ER due to PSEN1 can elevate lysosomal pH, thereby reducing their degradation capacity. Notably, this issue may be partially rescued by blocking ryanodine receptors or inositol 1, 4, 5-triphosphate receptors (IP3R), which are key channels regulating cellular calcium homeostasis and mediating calcium exchange between lysosomes and mitochondria. While the precise mechanism through which PSEN1 contributes to lysosomal deacidification remains incompletely understood, there is a consensus that elevated pH impairs the degradation of proteotoxic metabolites, such as APP-β CTF and Aβ peptides, promoting the progression of AD [232].

Lysosomes contain several hydrolases, with cathepsins (B, C, D, E, F, G, H, K, L, O, S, V, W, and X) being the most abundant. These enzymes are optimally active at low pH levels and degrade both endogenous and internalized exogenous protein completely into amino acids [233]. In AD, Cathepsin B, a cysteine protease, has been reported to exhibit β-secretase-like activity, cleaving wild-type human APP more efficiently than BACE1, thus contributing to AD pathogenesis. Conversely, CTSB also plays a protective role by degrading Aβ peptides and limiting their accumulation in neuronal cells [234]. Cystatin C, an endogenous cysteine protease inhibitor, modulates CTSB activity and has been shown to reduce Aβ load in AD mice [235]. Genetic ablation of Cystatin C results in decreased formation of intraneuronal Aβ plaques, improved cognitive deficits, and reduced premature death in an AD animal model [236]. Additionally, Cathepsin D (CTSD), an aspartic protease, has also been implicated in Aβ peptide clearance in vitro. Mutations in the CTSD gene, such as the A58V variant, are associated with abnormal processing of Aβ and tau [237]. Moreover, genetic deletion of CTSD leads to elevated levels of both Aβ42 and Aβ40 in the brain, further supporting its neuroprotective role in AD [238]. Another lysosomal aspartic protease, Cathepsin E (CTSE), shows increased expression in the cortex of the AD patient and the hippocampus of 6-month-old APP knock-in mice [239]. Pharmacological inhibition of CTSE has been shown to improve memory function, reduce Aβ accumulation, and alleviate neuroinflammation in APP KI mice, suggesting that CTSE could be a potential therapeutic target. Furthermore, Cathepsin L (CTSL), a cysteine protease, has demonstrated a protective role in AD. Both pharmacological inhibition and genetic knock-out of CTSL have been shown to mitigate Aβ42-induced damage to the nuclear lamina and the cleavage of lamin B1 [240]. These findings highlight the multifaceted roles of lysosomal cathepsins in AD pathology. Although some studies have reported contradictory findings regarding the degradation of Aβ peptides, further research is essential to optimize drug delivery systems and formulation for stability, bioavailability, and brain-specific targeting to achieve effective therapeutic outcomes.

**Table 2:** Potential small molecule inhibitor targeting ER stress in preclinical and clinical studies.

**Molecule**	**Primary Targets/Mechanism**	**Major Disease Model(s)**	**Experimental Evidence**	**References**
HA15,	Bip/GRP78 inhibitor	Osteosarcoma	In vivo, in vitro	[241]
OSU-03012		Osteosarcoma		[242]
IKMS		Breast cancer		[243]
KP1339		Colon cancer		[244]
STF-083010	IRE1α inhibitor	Multiple myeloma	In vitro, in vivo	[245]
B-109		Chronic lymphocytic leukemia		[246]
4µ8C		Colon cancer		[247]
Compound 18		Tumor		[248]
MKC8866		Reduce inflammation, suppresses NLRP3 activation		[249]
GSK2606414 & GSK2656157	PERK inhibitor	Diabetes, neurodegeneration, and colon cancer	In vitro, in vivo	[250,251]
Small molecule 42215		Suppresses elF2α phosphorylation in colorectal cancer		[252]
Tauroursodeoxycholic acid (TUDCA)	ER stress inhibitor	Diabetes, cardiovascular disease, and hypertension	In vitro, in vivo	[253]
Ursodeoxycholic acid (UDCA)		Peritoneal fibrosis, hepatobiliary diseases		[254]
Compound 11 (11a)	AEP inhibitor	Reduced Aβ 40–42, truncated tau, and pTau	In vivo, 3XFAD, and Tau-P301S	[24,255]
E64FC26 (EFC)	PDIs inhibitor	Rheumatoid arthritis and cancer	In vitro, in vivo	[195]
Dantrolene	RyR3 antagonist	Reduced enhanced Ca^2+^ cytosol level in 3xTg-AD mice	In vivo, in vitro	[256]
Rycal S107	Stabilizes calstabin 2 complex, inhibits calpain	Prevented ER Ca^2+^ leakage in AD mouse model, and tau phosphorylation	In vivo, in vitro	[257]
Aducanumab	Ameliorates calcium overload	Clear Aβ in Tg2576 mice	In vivo	[258]
Memantine	Inhibits excessive Ca^2+^ efflux	NMDR receptor antagonist inhibits Aβ oligomers	In vivo	[259]
Hyperoside (HYP)	Regulates RyR2 and ER Ca^2+^	Improved memory impairment and cognitive functions in APP/PS1 mice	In vivo	[260]
PTPIP51	Restores Ca^2+^ homeostasis and autophagy	Modulated MERCS mediated autophagy	In vitro	[261]
Pridopidine	MERCS-resident S1R chaperone	Ca^2+^ homeostasis at MERCS	In vivo, in vitro	[261]
Xestospongin B and C	IP3R inhibitor	Modulated MERCS mediated autophagy	In vivo	[261]
MCUi4/MCUi11	Inhibits mitochondrial Ca^2+^ accumulation in matrix	Insp3-dependent mitochondrial Ca^2+^ uniporter	In vivo	[262]
Metformin	Prevent excessive Ca^2+^ shuttling	Neuroprotective in AD/MCI patients	In vivo	[263]
Sulforaphane (SFN), Avmacol	Improves ER mitochondrial interaction via Nrf2	Neuroprotective agent in AD	In vivo	[264]
Trolox	Prevents ROS production, inhibits GSK3	Stabilizes MERCS and improved AD phenotype	In vivo	[265]
Luteolin	IP3Rs-dependent pathways response	Induces Ca^2+^ ATPase 2a (SERCA2a) activity in AD	In vitro	[266]
Urolithin A	Attenuated Aβ deposition, microgliosis, and astrocytosis in the cortex and hippocampus	Improves cognition and enhances neurogenesis	In vivo	[267]

## 5. Conclusions and Future Prospects

Alzheimer’s disease is increasingly recognized as a multifactorial disorder characterized by overlapping disturbances in proteostasis and organelle interactions. On one hand, there is significant evidence that molecular chaperones, including Hsps and their co-chaperone such as small HSPs, Hsp60, Hsp70, Hsp90, CHIP, and both ER and mitochondrial chaperones, play a crucial role in preventing early misfolding, aggregation, and propagation of Aβ and hyperphosphorylation of tau. On the other hand, dysfunctions in mitochondria and the ER, especially at MERCS, compromise calcium buffering, redox balance, and metabolic flexibility, potentially leading to synaptic failure and neuronal cell death. Importantly, these two aspects of AD pathology are interconnected; impaired chaperone networks exacerbate organelle stress, while mitochondrial depolarization and chronic ER stress further overwhelm the proteostasis machinery.

**Figure 3:**
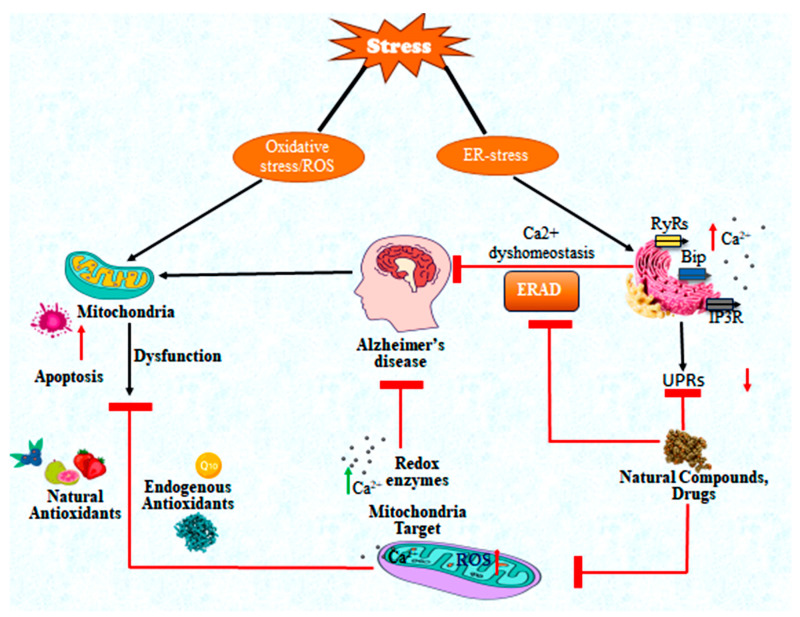
This schematic illustrates the roles of the ER and mitochondria in AD. Both organelles become primary targets under various cellular stress conditions. In AD, oxidative stress and ER stress disrupt the interaction between the ER and mitochondria, which impairs crucial pathways such as the regulation of ROS, ERAD, and UPRs. Targeting these pathways therapeutically, especially through the use of natural compounds and antioxidants, may help to improve cellular dysfunctions related to AD, particularly in early stages of the disease. The symbols (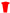) indicate the expression of upregulated proteins, (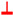) denotes inhibitory activity of the compounds.

Therapeutic innovation will require a comprehensive strategy that focuses on the following three key areas: (Ι) re-engaging chaperone and co-chaperone activity to prevent toxic protein aggregation, (ΙΙ) restoring mitochondrial and ER homeostasis to stabilize cellular energy and calcium signaling, and (ΙΙΙ) targeting the interface of MERCS where these pathways intersect (Figure 3). Recently, the FDA approved monoclonal antibody therapies, including IgGγ1 aducanumab, donanemab, and laecanemab, which have demonstrated clinical efficacy in phase III trials for AD. Numerous antioxidants, such as natural polyphenolic compounds, vitamins, flavonoids, and metal chelators, have shown protective roles both in vivo and in vitro [268]. These compounds have been effective in reducing ROS levels, ROS-induced toxicity, and inflammatory cytokines. However, only a limited number of studies have reported that these antioxidants can efficiently cross the blood–brain barrier and effectively prevent the ROS production. The exact mechanisms are not yet fully understood, and further investigations are needed to explore the antioxidant pathways involved [269]. In addition, several preclinical studies have investigated small-molecule chaperone modulators, mitophagy enhancers, and ER stress inhibitors, along with advancements at the molecular and cellular level, such as gene therapy, targeted protein degradation, and mitochondrial transplantation. A promising emerging class of molecular chaperones, known as CLABPs, has shown potential in inhibiting the aggregation of misfolded amyloid peptides and proteins.

A systematic and quantitative approach to studying the proteomics of MERCS under both physiological and disease conditions could uncover context-specific vulnerabilities and enhance rational drug design. However, the field must address methodological inconsistencies, adopt stage-specific disease models, and establish standardized measures for mitochondrial Ca^2+^ dynamics, chaperone function, and MERCS integrity. By integrating the study of proteostasis with interactions between mitochondria and the ER, future translational research has the potential to lead to significant disease-modifying interventions. These interventions could delay the onset of symptoms, slow disease progression, and ultimately alleviate the substantial personal and societal burden of AD.

## Data Availability

No new data were created or analyzed in this study.

## Abbreviations

AD	Alzheimer’s disease
Aβ	Amyloid-β
ALS	Amyotrophic lateral sclerosis
APP	Amyloid precursor protein
BACE1	β-Site APP cleaving enzyme-1
CLABPs	Chaperone-like amyloid-binding proteins
CSF	Cerebrospinal fluid
ETC	Electron transport chain
ER	Endoplasmic reticulum
ERAD	ER associated degradation
ERMES	ER-mitochondria encounter structure
FAD	Familial AD
FTD	Frontotemporal dementia
Grp75	Glucose-regulated protein 75
Hsps	Heat shock proteins
HD	Huntington’s disease
IP3Rs	inositol 1,4,5-trisphosphate receptors
LBD	Lewy-body dementia
LOAD	Late-onset Alzheimer’s disease
LTP	Long-term potentiation
MAMs	Mitochondria-associated ER membranes
MERCS	Mitochondrial–ER contact sites
MCU	Mitochondrial calcium uniporter
mPTP	Mitochondrial permeability transition pore
NBD	Nucleotide binding domain
NFTs	Neurofibrillary tangles
PACS2	Phosphor-furin acidic cluster sorting protein 2
PDI	Protein disulfide isomerase
PD	Parkinson’s disease
PolyQ	Polyglutamine
RyRs	Ryanodine receptors
ROS	Reactive oxygen species
S1R	Sigma-1 receptor
SBD	Substrate binding domain
SOCE	Store-operated Ca2+ entry
STIM	Stromal interaction molecule
TEM	Transmission electron microscopy
TLR	Toll like receptors
TRAP1	Tumor necrosis factor receptor-associated protein 1
TRPC	Transient receptor potential canonical
Ub	Ubiquitin
UPR	Unfolded protein responses
UPS	Ubiquitin–proteasome system
VD	Vascular dementia
